# Effect of multidrug therapy on the prognosis of *Mycobacterium avium* complex pulmonary disease

**DOI:** 10.1038/s41598-024-55135-0

**Published:** 2024-02-23

**Authors:** Atsuhito Ushiki, Shunnosuke Tanaka, Miwa Yamanaka, Jumpei Akahane, Yuichi Ikuyama, Masamichi Komatsu, Kei Sonehara, Takashi Ichiyama, Yosuke Wada, Kazunari Tateishi, Yoshiaki Kitaguchi, Masayuki Hanaoka

**Affiliations:** First Department of Internal Medicine, Shinshu University School of Medicine, 3-1-1 Asahi, Matsumoto, 390-8621 Japan

**Keywords:** Antimicrobials, Respiratory tract diseases

## Abstract

Multidrug therapy for *Mycobacterium avium* complex pulmonary disease (MAC-PD) results in negative sputum cultures. However, the prognostic value of this treatment approach remains unclear. This study aimed to clarify whether multidrug therapy reduces the incidence of events related to MAC-PD and improves the mortality rate. Patients who met the diagnostic criteria for MAC-PD at our hospital between 2003 and 2019 were retrospectively evaluated using medical records. Events related to MAC-PD were defined as hospitalisation for haemoptysis or respiratory infection and the development of chronic respiratory failure. There were 90 and 108 patients in the multidrug and observation groups, respectively. The median observation period was 86 months. Intergroup differences in body mass index, proportion of patients with cavities, and erythrocyte sedimentation rate were not significant. However, the observation group was older with a higher mean age (multidrug group: 62 years, observation group: 69 years; P < 0.001) and had a higher proportion of male patients (multidrug group: 13/90 [14.4%], observation group: 35/108 [32.4%]; P < 0.01). Furthermore, intergroup differences in the incidence of events related to MAC-PD (multidrug group: 26.69/1000 person-years, observation group: 25.49/1000 person-years), MAC-PD-associated mortality rate (multidrug group: 12.13/1000 person-years, observation group: 12.74/1000 person-years), and total mortality (multidrug group: 24.26/1000 person-years, observation group: 29.50/1000 person-years) were not significant. Many patients relapse even after multidrug therapy, and our findings suggest that multidrug therapy has no effect in preventing the onset of respiratory events or prolonging life expectancy.

## Introduction

Non-tuberculous mycobacteria are environmentally ubiquitous. Pulmonary non-tuberculous mycobacterial disease can be caused by inhaling mycobacteria from the environment. In recent years, an increasing number of cases of pulmonary non-tuberculous mycobacterial disease have been reported worldwide^1–3^. There are over 190 species of non-tuberculous mycobacteria^4^; however, most pulmonary non-tuberculous mycobacterial disease cases are attributable to the *Mycobacterium avium* complex (MAC)^3,5,6^.

The 5-year and 10-year all-cause mortality rates in patients with MAC pulmonary disease (MAC-PD) have been reported as 23.9% and 46.5%, respectively, and the corresponding MAC-specific mortality rates were 5.4% and 15.7%^7^. Multidrug therapies, including clarithromycin, can yield bacteriological sputum culture conversion in patients with MAC-PD^8,9^. However, few studies have examined whether it can reduce hospitalisation and respiratory failure related to MAC-PD or mortality. Therefore, this study examined whether multidrug therapy reduces the incidence of events related to MAC-PD and improves the prognosis of the affected patients.

## Methods

### Study design and patients

This retrospective study was conducted by evaluating patients’ medical records. Patients who were admitted to our hospital between 2003 and 2019, who met the 2020 diagnostic criteria established by the American Thoracic Society/European Respiratory Society/European Society of Clinical Microbiology and Infectious Diseases/Infectious Diseases Society of America, and for whom cultures were positive for MAC were included in the study^10^. The exclusion criteria were as follows: patients who had undergone surgery for MAC-PD, those with additional microbial infections other than MAC disease, and those with missing data required for the score assessment, which is a scoring system involving calculations based on the body mass index, age, presence of cavities, an elevated erythrocyte sedimentation rate, and sex, and reflects disease progression and mortality^11,12^. The included patients were divided into two groups. The multidrug therapy group included patients who received clarithromycin, ethambutol, and rifampicin for at least 1 year (including patients concomitantly treated with quinolones or aminoglycosides). The observation group included all the remaining patients.

The following three outcomes were analysed:

1. MAC-associated events, defined as hospitalisation for haemoptysis or respiratory infection and the onset of chronic respiratory failure;
2. MAC-specific mortality, defined as death due to haemoptysis, respiratory infection, or respiratory failure;
3. all-cause mortality.

We also conducted a prognosis analysis of patients who underwent multiple courses of treatment, classifying them according to the initial treatment.

### Statistical analysis

Continuous and ordinal variables were expressed as medians and interquartile ranges, and groups were compared using Mann − Whitney *U* tests. Categorical variables were expressed as frequencies and percentages, and groups were compared using χ^2^ tests or Fisher's exact probability test. The incidence of MAC-PD-associated events, MAC-specific mortality, and all-cause mortality were expressed as the number of patients per 1000 person-years. Risk ratios and 95% confidence intervals (CI) were calculated. Kaplan–Meier curves were used to assess survival. The median values (95% CI) were presented, and the Gray test was used to compare MAC-PD-associated events and MAC-specific mortality between groups. The log-rank test was used to compare all-cause mortality. Factors predicting MAC-PD-associated events, MAC-specific mortality, and all-cause mortality were examined using single variable and multivariable Cox regression analyses. P-values < 0.05 were considered statistically significant. All statistical analyses were performed using EZR^13^, a modified version of the R commander designed to add statistical functions frequently used in biostatistics.

### Ethical considerations

This study was approved by the medical ethics committee of Shinshu University School of Medicine (approval No. 4575). All methods were performed in accordance with the relevant guidelines and regulations. Informed consent was obtained from all patients through an opt-out process.

## Results

Overall, 198 patients were included in this study, of whom 90 were included in the multidrug therapy group and 108 in the observation group. Among the patients in the multidrug therapy group, 23 were treated concurrently with aminoglycosides, 6 were treated concurrently with quinolones, and 3 were treated concurrently with both aminoglycosides and quinolones. Among the patients in the multidrug therapy group, 86 received one course of treatment and 4 underwent two courses of treatment. Of the 108 patients in the observation group, 20 had a treatment duration of less than 1 year, with 19 discontinuing treatment due to adverse events, and 1 discontinuing treatment due to interruption of outpatient visits. The median treatment duration for these 20 patients was 35.5 days (interquartile range, 19–90.75). The patient characteristics are shown in Table 1. The observation group was significantly older than the multidrug therapy group, and the total protein level in the observation group was significantly lower than that in the multidrug therapy group. The two groups had no significant differences in the mycobacterial species, diagnostic specimens, and sputum smear positivity for acid-fast bacillus. The body mass index, age, cavitary erythrocyte sedimentation rate, and sex (BACES) score in the observation group was 2 (range, 1–3), which was significantly higher than the score of 1 (range, 1–2.75) in the multidrug therapy group. The observation group had significantly higher proportions of patients aged 65 years and older and male patients, which contributed to the higher BACES score. Table 2 shows the incidence of MAC-associated events and MAC-specific mortality and all-cause mortality rates per 1000 person-years in the two groups. There were no significant differences in any of the outcomes between the groups.

**Table 1:** Patient characteristics.

Characteristic	Multidrug therapy group (n = 90)	Observation group (n = 108)	P
Age, years	62 (56–69)	69 (62–75)	< 0.001
BMI, kg/m^2^	19.4 (17.2–21.2)	18.7 (17.7–20.6)	0.79
WBCs, /μL	5765 (4803–6935)	5715 (4610–7540)	0.82
Lymphocytes, /μL	1570 (1200–2000)	1425 (1000–1900)	0.30
CRP, mg/dL	0.11 (0.03–0.46)	0.10 (0.05–0.48)	0.61
ESR, mm/H	18.5 (10–38.8)	18.5 (10–31)	0.52
TP, g/dL	7.5 (7.1–7.9)	7.3 (6.9–7.6) (n = 102)	< 0.01
Albumin, g/dL	4.1 (3.9–4.4) (n = 88)	4.0 (3.8–4.3) (n = 101)	0.38
Mycobacterial species			> 0.99
*M. avium*	72 (80.0)	87 (80.6)	
*M. intracellulare*	13 (14.4)	15 (13.9)	
*M. avium* + *M. intracellulare*	5 (5.6)	6 (5.6)	
Diagnostic specimen			0.73
Sputum	44 (48.9)	49 (45.5)	
Bronchial lavage fluid	46 (51.1)	59 (54.6)	
Acid-fast bacillus smear positivity of sputum	22(24.4)	23 (23.1)	0.61
Time from diagnosis to treatment, months	16 (2–68)		
Follow-up duration, person-years	824.3	784.8	
BACES score	1 (1–2.75)	2 (1–3)	< 0.01
BMI < 18.5 kg/m^2^	35 (38.9)	48 (44.4)	0.52
Age ≥ 65 years	35 (38.9)	69 (63.9)	< 0.001
Cavity on chest image	18 (20.0)	23 (21.3)	0.96
Elevated ESR	44 (48.9)	52 (48.1)	> 0.99
Sex			< 0.01
Male	13 (14.4)	35 (32.4)	
Female	77 (85.6)	73 (67.6)	

*BACES* body mass index, age, cavitary, erythrocyte sedimentation rate, and sex, *BMI* body mass index, *CRP* C-reactive protein, *ESR* erythrocyte sedimentation rate, *TP* total protein, *WBCs* white blood cells.

**Table 2:** The incidence of MAC-pulmonary disease-associated events, MAC-specific mortality, and all-cause mortality.

	Multidrug therapy group (n = 90)	Observation group (n = 108)	Relative risk (95% CI)
Incidence of MAC-PD associated events, /1000 person-years			
All events	26.69	25.49	1.05 (0.61–1.79)
Hospitalisation for haemoptysis	4.85	6.37	0.76 (0.23–2.47)
Hospitalisation for respiratory infections	12.13	15.29	0.79 (0.38–1.68)
Chronic respiratory failure	9.71	3.82	2.54 (0.78–8.28)
MAC-specific mortality, /1000 person-years	12.13	12.74	0.92 (0.44–2.08)
All-cause mortality, /1000 person-years	24.26	29.50	0.82 (0.48–1.40)

*CI* confidence interval, *MAC* *Mycobacterium avium* complex.

**Figure 1:**
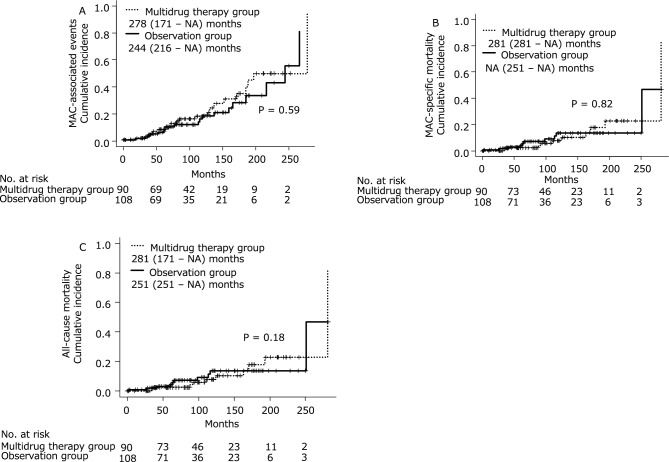
(**A**) MAC-associated events. The median time to MAC-associated events was 278 months in the multidrug therapy group and 244 months in the observation group, with no significant intergroup differences. (**B**) MAC-specific mortality. The median time to MAC-specific death was 281 months in the multidrug therapy group and NA in the observation group, with no significant intergroup difference. (**C**) All-cause mortality. The median time to all-cause death was 281 months in the multidrug therapy group and 251 months in the observation group, with no significant intergroup difference. *MAC Mycobacterium avium* complex, *NA* not available.

Figure 1 shows the Kaplan–Meier curves for the cumulative incidence of MAC-PD-associated events and cumulative MAC-specific mortality, and all-cause mortality. The median time to MAC-PD-associated events in the multidrug therapy and observation groups was 278 and 244 months, respectively. The corresponding median times to MAC-specific death were 281 months and not available (NA). The median time to all-cause death was 281 and 251 months in the multidrug therapy and observation groups, respectively. There were no significant differences in any of the outcomes between the two groups.

Table 3 shows the results of the unadjusted and adjusted Cox regression analyses. Considering the number of patients, events, and deaths, two explanatory variables were selected: BACES score and multidrug therapy. The two explanatory variables were analysed using the forced entry method for the multivariable analysis. The BACES score significantly predicted all outcome measures, including MAC-associated events, MAC-specific mortality, and all-cause mortality. However, multidrug therapy was not associated with any of the outcomes.

**Table 3:** Unadjusted and adjusted Cox regression analyses of the risk factors for each MAC-associated event, MAC-specific mortality, and all-cause mortality.

	Unadjusted analysis		Adjusted analysis	
	Hazard ratio (95% CI)	P	Hazard ratio (95% CI)	P
MAC-associated event				
BACES score	1.51 (1.18–1.94)	< 0.01	1.51 (1.18–1.94)	< 0.01
Multidrug therapy	1.01 (0.55–1.87)	0.97	1.02 (0.55–1.88)	0.96
MAC-specific mortality				
BACES score	2.21 (1.51–3.24)	< 0.001	2.21 (1.51–3.32)	< 0.001
Multidrug therapy	0.90 (0.37–2.17)	0.82	0.84 (0.33–2.12)	0.71
All-cause mortality				
BACES score	1.88 (1.46–2.42)	< 0.001	1.86 (1.45–2.39)	< 0.001
Multidrug therapy	0.66 (0.36–1.21)	0.18	0.71 (0.38–1.33)	0.28

*BACES* body mass index, age, cavitary, erythrocyte sedimentation rate, and sex, *CI* confidence interval, *MAC Mycobacterium avium* complex.

## Discussion

Multidrug therapy has been shown to reduce all-cause mortality in cases of pulmonary non-tuberculous mycobacterial disease^14–16^. However, these studies included MAC-PD and other pulmonary non-tuberculous mycobacterial diseases. For example, in studies that include infection due to bacterial species with relatively favourable treatment responses, such as *Mycobacterium kansasii*^17^, or relatively poor treatment responses, such as *Mycobacterium abscessus*^18^, the potential improvement in mortality rates through therapeutic interventions may differ from those in studies of MAC-PD alone. In this study which focused specifically on MAC-PD, multidrug therapy was not associated with decreased all-cause mortality. When comparing the two bacterial species within the MAC, *M. intracellulare* infections have been reported to have a worse prognosis than *M. avium* infections^14,19^. In this study, the number of patients with *M. intracellulare* infection was relatively small; therefore, the two bacterial species were not compared. However, there were no differences in the proportions of the two bacterial species between the multidrug therapy and observation groups. Furthermore, previous studies have also included patients who did not receive adequate treatment according to the current guidelines^10^, which involve administering a combination of three or more drugs, including macrolides and ethambutol, for at least 1 year after achieving negative sputum cultures.

In this study, multidrug therapy involved a treatment regimen containing clarithromycin, ethambutol, and rifampicin. Azithromycin is considered to have better tolerability than clarithromycin; however, there is no significant difference in efficacy between the two drugs^10^. In Japan, owing to medical regulations, azithromycin must be administered after clarithromycin treatment. Therefore, in this study, patients receiving macrolide therapy were specifically those who were administered clarithromycin.

The guidelines recommend a treatment duration of 1 year or more after sputum culture conversion to negative. However, in this study, over 50% of the cases were diagnosed using bronchial lavage fluid, as there was no expectoration of sputum. These cases without sputum expectoration could not be assessed for sputum culture conversion to negative, and as a result, the treatment duration is not specified in the guidelines. Therefore, this study defined the total treatment period as 1 year for the multidrug therapy group. To standardise the treatment duration, patients diagnosed based on sputum were also included in the multidrug therapy group, regardless of sputum culture conversion, if they received treatment for 1 year.

In this study, multidrug therapy was not associated with reduced overall mortality, MAC-associated events, or MAC-specific mortality. To our knowledge, no previous studies have investigated the effects of multidrug therapy on MAC-associated events or MAC-specific mortality. Furthermore, the BACES score is associated with all-cause mortality and death related to respiratory diseases and infections, treatment responsiveness, and disease progression^11,12^. In our study, patients in the observation group had a higher BACES score than those in the multidrug therapy group; however, the event and mortality rates did not differ between the two groups. These results suggest that multidrug therapy does not decrease the incidence of MAC-associated events or mortality.

Contrary to the findings of our study, a study by Kim et al.^20^ found that prognosis of MAC-PD was better in patients who received multidrug therapy. The results of the two studies may have differed because of differences in disease severity, such as the extent of bronchiectasis and systemic manifestations. Our study included a higher proportion of patients with lower BACES scores and relatively mild disease, such as those with negative sputum smears. Even without treatment, many of these patients are likely to have had a favourable prognosis.

The rate of negative conversion of sputum culture achieved with combination therapy is only 60–90%, which is suboptimal^8,9^. Relapse and reinfection are rare after achieving negative sputum culture^20,21^, which may have contributed to the findings of our study. A previous study demonstrated improved quality of life associated with treating pulmonary non-tuberculous mycobacterial disease^22^. A recent study evaluating the effectiveness of multidrug therapy has emphasised the importance of microbiological assessments and considering the quality of life^23^. Major symptoms such as severe fatigue are associated with markedly decreased quality of life and can also be key factors in starting multidrug therapy^10^. Therefore, the use of multidrug therapy to improve quality of life should be considered. When evaluating the improvement in the quality of life of patients with MAC-PD, it is essential to use established assessment tools^24^, such as St. George’s Respiratory Questionnaire, which has been validated for assessing the quality of life of patients with MAC-PD^25^.

Our study has some limitations. First, the treatments were not fully compliant with the guidelines because some patients did not receive treatment for 12 months after sputum culture conversion, and the recommended dose of antimicrobial agents was not consistently maintained. Second, susceptibility to clarithromycin was not confirmed in some cases, raising the possibility of MAC-PD caused by clarithromycin-resistant strains. Third, many cases were lost to follow-up within 5–10 years of the start of observation, leading to premature termination of the study. Fourth, the number of patients is relatively small, and this is also a single-centre study, so the generalisability may be limited.

Our study showed that multidrug therapy with clarithromycin, ethambutol, and rifampicin for MAC-PD may not reduce the incidence of hospitalisation events, respiratory failure related to MAC-PD, or mortality.

## Data Availability

The datasets generated during and/or analysed during the current study are not publicly available due to ethical restrictions but are available from the corresponding author on reasonable request.
